# Adult Protective Services Study on the Impact of COVID-19

**DOI:** 10.1093/geroni/igab046.330

**Published:** 2021-12-17

**Authors:** Karen Roberto, Pamela Teaster, Jyoti Savla, Imogen Fua, Karl Urban, Emily Hoyt

**Affiliations:** 1 Virginia Tech, Blacksburg, Virginia, United States; 2 Virginia Tech, BLACKSBURG, West Virginia, United States; 3 WRMA, Inc., Rockville, Maryland, United States; 4 WRMA, Inc., Arlington, Virginia, United States

## Abstract

The purpose of study was to explore changes in implemented of APS programs in response to the COVID-19 pandemic. This three-phase study began with telephone interviews with eight state-level APS administrators of early phase “hot-spots” that were used to inform questions for the Phase II national survey of state APS programs. Forty-seven states completed an on-line survey on the effects of the pandemic on work and workload, policy and practice, staff, partnerships, and preparedness. Phase III interviews with 7 local APS programs provided an “on-the-ground” view of challenges of meeting clients’ needs. Although most APS programs reported fewer reports of adult maltreatment than before the pandemic, the stress and disruption of COVID-19 required new work arrangements, to which programs adapted technological and managerial support to care for the needs of both APS staff and the clients they served. Finding provide insight for policy and planning requirements for future catastrophic events.

